# Dynamic Changes in Pre- and Postoperative Levels of Inflammatory Markers and Their Effects on the Prognosis of Patients with Gastric Cancer

**DOI:** 10.1007/s11605-020-04523-8

**Published:** 2020-02-03

**Authors:** Jian-Xian Lin, Zu-Kai Wang, Ying-Qi Huang, Jian-Wei Xie, Jia-Bin Wang, Jun Lu, Qi-Yue Chen, Mi Lin, Ru-Hong Tu, Ze-Ning Huang, Ju-Li Lin, Chao-Hui Zheng, Chang-Ming Huang, Ping Li

**Affiliations:** 1 Department of Gastric Surgery, Fujian Medical University Union Hospital, Fuzhou, Fujian Province China; 2 Department of General Surgery, Fujian Medical University Union Hospital, Fuzhou, Fujian Province China; 3 Key Laboratory of Ministry of Education of Gastrointestinal Cancer, Fujian Medical University, Fuzhou, Fujian Province China; 4 Fujian Key Laboratory of Tumor Microbiology, Fujian Medical University, Fuzhou, Fujian Province China

**Keywords:** Gastric Cancer, Systemic inflammatory response, Prognosis

## Abstract

**Background:**

Whether the change of the pre- and postoperative systemic inflammatory response (SIR) levels will affect the prognosis of gastric cancer (GC) is unclear. We aimed to investigate the dynamic changes in the pre- and postoperative SIR and their prognostic value for GC.

**Methods:**

The clinicopathological data from 2257 patients who underwent radical gastrectomy between January 2009 and December 2014 at Fujian Medical University Union Hospital (FMUUH) were analyzed. Perioperative SIR changes were reported as changes in the lymphocyte-monocyte ratio (LMR), neutrophil-lymphocyte ratio (NLR), platelet-lymphocyte ratio (PLR), and systemic immune-inflammation index (SII).

**Results:**

The SIR levels showed different trends from postoperative months 1 to 12. Multivariate analysis showed that preoperative (pre)-LMR was an independent predictor for the prognosis (*P* = 0.024). The postoperative 12-month (post-12-month) LMR predicted the 5-year overall survival (OS) rate with the highest accuracy (areas under the curve [AUC] 0.717). Patients were divided into four groups according to the optimal cutoff of the preoperative and post-12-month LMR: high pre-LMR to high postoperative (post)-LMR group, high pre-LMR to low post-LMR group, low pre-LMR to high post-LMR group, and low pre-LMR to low post-LMR group. The survival analysis showed 5-year OS rate was significantly higher in patients with high post-12-month LMR than in patients with low post-12-month LMR, regardless of pre-LMR levels (81.6% vs. 44.2%, *P* < 0.001). The prognostic accuracy was significantly improved by incorporating the post-12-month LMR in the tumor-node-metastasis (TNM) staging system (*P* = 0.003).

**Conclusions:**

The remeasurement of LMR at post-12-month is helpful in predicting the long-term survival of GC.

**Electronic supplementary material:**

The online version of this article (10.1007/s11605-020-04523-8) contains supplementary material, which is available to authorized users.

## Introduction

Gastric cancer (GC) is one of the most common malignancies of the digestive system; it is the 5th most common malignancy and the 3rd leading cause of cancer-related death.^1^ In the past few decades, significant progress has been achieved in using diagnostic techniques and treatment regimens to improve the survival of patients with GC.^2–6^ However, the survival of these patients could be further improved, particularly through the discovery of widely available and inexpensive biomarkers for the diagnosis, determination of the prognosis, determination of the requirement for adjuvant therapy, and monitoring of therapeutic responses. In recent years, some studies have explored the relationship between different inflammatory markers and the prognosis of patients with GC.^7–10^ The lymphocyte-to-monocyte ratio (LMR), neutrophil-to-lymphocyte ratio (NLR), platelet-lymphocyte ratio (PLR), and systemic immune-inflammation index (SII) are the most easily obtained inflammatory markers and can be obtained from complete blood count (CBC) testing, which is a convenient method for dynamic preoperative and postoperative repeated measurements.^11–14^ As potential markers for predicting the prognosis and guiding the treatment of patients with GC, they reflect the complex interactions between the local immune response and systemic inflammatory response (SIR) in the tumor microenvironment.^15^ However, previous studies have mostly been limited to exploring the relationship between the preoperative (pre)-SIR and the prognosis. Following surgery and adjuvant chemotherapy, postoperative (post)-SIR levels may differ from preoperative levels. In this study, we aimed to longitudinally investigate and characterize the SIR from the preoperative period through multiple time points in the postoperative period to examine the dynamic changes in perioperative LMR, NLR, PLR, and SII and to investigate whether changes in the SIR would confer a difference in the overall survival (OS).

## Materials and Methods

### Patients

This study retrospectively analyzed the clinicopathological data from patients undergoing radical gastrectomy at Fujian Medical University Union Hospital (FMUUH) from January 2009 to December 2014. The following inclusion criteria were established: (1) number of examined nodes > 15 and (2) no evidence of distant metastasis. Patients were excluded if they (1) had received neoadjuvant therapy, (2) had not undergone a routine blood examination before surgery, or (3) had incomplete/inaccurate clinical or pathological information. Finally, 2257 patients were included in this study for the baseline analysis (Supplementary Fig. 1). The type of surgical resection and the extent of lymph node dissection were selected according to the guidelines of the Japanese Gastric Cancer Association.^16^ Six to eight cycles of adjuvant chemotherapy using 5-fluorouracil (5-FU)-based regimens (mostly oxaliplatin with either Xeloda or S-1) were recommended for the majority of patients with advanced GC. The postoperative pathological stage of the tumor was determined according to the eighth edition of the American Joint Committee on Cancer (AJCC) and Union for International Cancer Control (UICC) staging manual.^17^

### Definition of Pre- and Postoperative Inflammatory Markers

Patients routinely underwent blood testing during the 7 days before surgery. For postoperative blood testing, samples were routinely collected at multiple time points. The following periods were investigated: 21st to 45th postoperative days (POD) (defined as post-1-month), 46th to 75th POD (defined as post-2-month), 76th to 105th POD (defined as post-3-month), 106th to 135th POD (defined as post-4-month), 136th to 165th POD (defined as post-5-month), 166th to 270th POD (defined as post-6-month), and 271st POD and later (defined as post-12-month). If multiple blood test results were available in the same period, the result closest to each time point after surgery was recorded. For example, if one patient underwent postoperative blood tests at POD 342 and POD 390, the results from the test performed at POD 342 were recorded, because these results were closest to post-12-month (POD 360). Because of differences in physician follow-up preferences, differences in the tumor stages of patients in the cohort, and differences in patient compliance, not all patients underwent blood testing at each of these time points. The preoperative value of CBC-based markers of this study refers to the preoperative average value of the population with blood available at a specific postoperative time point, but not the average value of the baseline population. For example, there were 337 patients with blood available at post-6-month, the pre-LMR value at this time point is the average value of the pre-LMR of 337 patients, and at post-12-month, 157 patients underwent postoperative blood tests, and the pre-LMR value at this time point was the average LMR value of 157 patients. This ensures the consistency of the population before and after surgery at the corresponding time points, which may help to compare pre- and postoperative changes as well as to reduce errors. Candidate CBC-based inflammatory markers in our study included the LMR, NLR, PLR, and SII (platelets*NLR).

### Optimal Cutoff Values of Candidate CBC-Based Inflammatory Markers According to the X-Tile

The X-tile program (http://medicine.yale.edu/lab/rimm/research/software.aspx) was used to determine the optimal cutoff values of candidate CBC-based inflammatory markers for OS. X-tile plots present a new tool for the assessment of biological relationships between a biomarker and outcome and the discovery of population cut-points based on marker expression. A population is divided into different divisions based on every possible cutoff point. All possible divisions of the cutoff point are statistically assessed. Then, X-tile plots calculate *χ*^2^ values for every possible division of the population. The optimal cutoff value for survival was calculated by selecting the minimum *P* value with the maximum *χ*^2^ value.^18^ The cutoff values for the pre-LMR, NLR, PLR, and SII were 3.42, 3.87, 162.50, and 569.93, respectively, and the cutoff value of post-12-month LMR was 4.00, as determined by the X-tile software.

### Follow-Up Investigation

The postoperative follow-up evaluation generally consisted of clinical visits, laboratory testing, and computed tomography (CT) scans that were repeated every 3–6 months for 2 years, every 6–12 months from years 2 to 5, then annually thereafter. The survival time was recorded from the date of surgery to the last follow-up date, date of death, or date at the end of follow-up in the database (such as loss to follow-up or death due to other diseases).

### Statistical Analysis

Descriptive statistics were used to summarize cohort characteristics, and continuous variables are reported as means ± standard deviation (SD). The paired-sample *t* test was used to determine the statistical significance of changes in the preoperative and postoperative levels of inflammatory markers at each time point. Both the log-rank test and multivariate Cox proportional hazards regression models were used to analyze the relationship between serum inflammatory markers and OS. Variables that were statistically significant (*P* < 0.05) in the univariate analysis were entered into a multivariate Cox regression model. Survival estimates were reported as hazard ratios (HRs) with 95% confidence intervals (CIs). Areas under the curves (AUCs) were calculated to estimate the prognostic abilities of the preoperative and postoperative inflammatory markers as risk factors for OS. Statistical analyses were performed using SPSS 22.0 software (SPSS, Chicago, IL, USA) and R 3.6.0 software (The R Foundation for Statistical Computing, Vienna, Austria). All statistical tests were two-sided, and a *P* value < 0.05 was considered statistically significant.

## Results

### Baseline Characteristics

Of the 2257 patients with GC included in the study, 1698 (75.2%) were male and 559 (24.8%) were female, and the mean age was 60.9 ± 11.2 years. The majority had an American Society of Anesthesiologists (ASA) score of 1, with a mean body mass index (BMI) of 22.8 ± 8.8 kg/m^2^. In terms of disease characteristics, the majority of the tumors were located in the lower third of the stomach (42.1%), with a mean tumor size of 45.2 ± 25.1 mm. Total gastrectomy was performed in 1180 cases (52.3%), distal gastrectomy was performed in 1027 cases (45.5%), and proximal gastrectomy was performed in 49 cases (2.2%). Most patients were diagnosed with the undifferentiated histological type (77.1%), without vascular invasion (77.1%) or perineural invasion (83.6%). The distribution of TNM stages were as follows: 660 (29.2%) patients with stage I, 553 (24.5%) with stage II, and 1044 (46.3%) with stage III disease. More than half (52.7%) of the patients received postoperative adjuvant chemotherapy (485 patients lacked records of postoperative adjuvant chemotherapy). Regarding the CBC-based inflammatory markers, the mean LMR, NLR, PLR, and SII were 4.5 ± 2.1, 2.6 ± 2.2, 154.2 ± 79.1, and 636.6 ± 593.5, respectively. The baseline characteristics of this cohort are documented in Supplementary Table 1.

### Changes in Pre- and Postoperative CBC-Based Inflammatory Markers

**Fig. 1:**
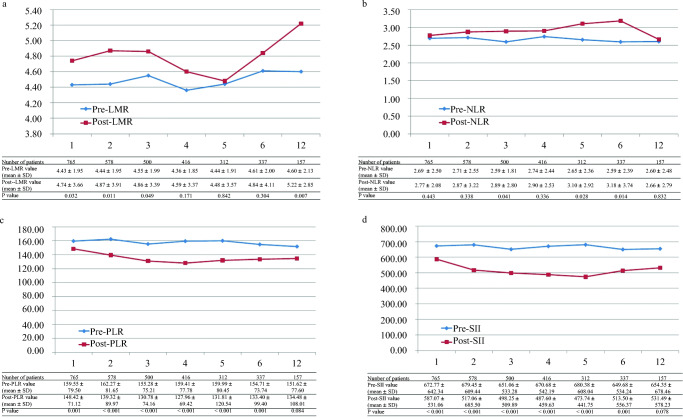
Changes of values in pre- and postoperative CBC-based inflammatory markers at multiple time periods. **a** The perioperative LMR changes. **b** The perioperative NLR changes. **c** The perioperative PLR changes. **d** The perioperative SII changes

We assessed the changes in LMR, NLR, PLR, and SII from the preoperative to the postoperative periods at multiple postoperative time points (Fig. 1). The number of patients with blood test results available for analysis in each period ranged from 157 at post-12-month to 765 patients at post-1-month. The post-LMR ranged from 4.5 ± 3.6 for patients at post-5-month to 5.2 ± 2.9 for patients at post-12-month. The LMR was higher at each postoperative time point than before surgery and showed an “up-down-up” trend postoperatively. At post-1, 2, 3, and 12 months, the difference in LMR between preoperative and postoperative assessments was statistically significant (all *P* < 0.05). The post-NLR, PLR, and SII also showed different trends over time respectively, but at post-12 month, only LMR was significantly higher than the preoperative values (Fig. 1).

### Effect of Preoperative Inflammatory Markers on Overall Survival

The median follow-up time of the entire cohort was 65.6 months (range from 1 to 117 months), with a 5-year OS rate of 67.5%. According to the univariate analysis, the four CBC-based inflammatory markers were all associated with OS (all *P* < 0.001). In addition, other variables, including age, BMI, ASA score, tumor location, tumor size, histological type, vascular invasion, perineural invasion, pathological tumor-node-metastasis (pTNM) stage, and adjuvant chemotherapy, were also associated with OS (all *P* < 0.05). In multivariate analyses, only the pre-LMR (*P* = 0.024) was an independent CBC-based inflammatory marker for OS. Other independent variables, including age, BMI, tumor location, tumor size, pTNM stage, and adjuvant chemotherapy, were associated with OS (all *P* < 0.05) (Table 1).

**Table 1:** Univariate and multivariate analyses of clinicopathological variables in relation to overall survival in patients undergoing radical gastrectomy for gastric cancer

Clinicopathological features	Univariate analysis	*P*	Multivariate analysis	*P*
	HR (95% CI)		HR (95% CI)	
Age				
< 60	Reference		Reference	
≥ 60	1.674 (1.443–1.941)	< 0.001	1.493 (1.257–1.772)	< 0.001
Sex				
Male	Reference			
Female	0.999 (0.849–1.175)	0.988		
BMI		< 0.001		0.004
BMI < 18.5	Reference		Reference	
18.5 ≤ BMI < 25	0.619 (0.501–0.765)	< 0.001	0.742 (0.585–0.940)	0.013
BMI ≥ 25	0.482 (0.365–0.636)	< 0.001	0.592 (0.433–0.810)	0.001
ASA score		0.010		0.406
1	Reference		Reference	
2	1.221 (1.054–1.414)	0.008	1.110 (0.941–1.309)	0.214
3	1.41 (0.980–2.028)	0.064	1.171 (0.768–1.787)	0.463
Tumor location		< 0.001		< 0.001
Upper	Reference		Reference	
Middle	0.901 (0.736–1.102)	0.310	0.874 (0.692–1.105)	0.262
Lower	0.611 (0.509–0.733)	< 0.001	0.744 (0.609–0.910)	0.004
Mixed	1.489 (1.205–1.839)	< 0.001	1.259 (0.995–1.592)	0.055
Tumor size (mm)				
< 50	Reference		Reference	
≥ 50	2.66 (2.303–3.073)	< 0.001	1.355 (1.146–1.604)	< 0.001
Histologic type				
Differentiated	Reference		Reference	
Undifferentiated	1.213 (1.018–1.445)	0.031	0.950 (0.778–1.161)	0.618
Vascular invasion				
Negative	Reference		Reference	
Positive	1.814 (1.560–2.111)	< 0.001	0.949 (0.783–1.149)	0.589
Perineural invasion				
Negative	Reference		Reference	
Positive	1.782 (1.507–2.106)	< 0.001	1.023 (0.827–1.265)	0.835
pTNM stage		< 0.001		< 0.001
I	Reference		Reference	
II	3.161 (2.296–4.352)	< 0.001	2.846 (2.021–4.007)	< 0.001
III	10.648 (8.037–14.108)	< 0.001	8.126 (5.932–11.132)	< 0.001
Adjuvant chemotherapy*				
No	Reference		Reference	
Yes	1.365 (1.169–1.593)	< 0.001	0.802 (0.682–0.943)	0.008
Pre-LMR				
< 3.42	Reference		Reference	
≥ 3.42	0.649 (0.562–0.749)	< 0.001	0.810 (0.674–0.973)	0.024
Pre-NLR				
< 3.87	Reference		Reference	
≥ 3.87	1.636 (1.354–1.976)	< 0.001	1.218 (0.951–1.561)	0.119
Pre-PLR				
< 162.50	Reference		Reference	
≥ 162.50	1.613 (1.400–1.859)	< 0.001	1.176 (0.966–1.433)	0.107
Pre-SII				
< 569.93	Reference		Reference	
≥ 569.93	1.475 (1.282–1.697)	< 0.001	0.840 (0.686–1.028)	0.091

*CI* confidence interval, *HR* hazard ratio, *BMI* body mass index, *ASA* American Society of Anesthesiologists, *TNM* tumor-node-metastasis, *LMR* lymphocyte-monocyte ratio, *NLR* neutrophil-lymphocyte ratio, *PLR* platelet-lymphocyte ratio, *SII* systemic immune-inflammation index

*A total of 485 patients missing adjuvant chemotherapy

### Effect of Changes Between the Preoperative and Post-12-Month LMR on Prognosis

**Fig. 2:**
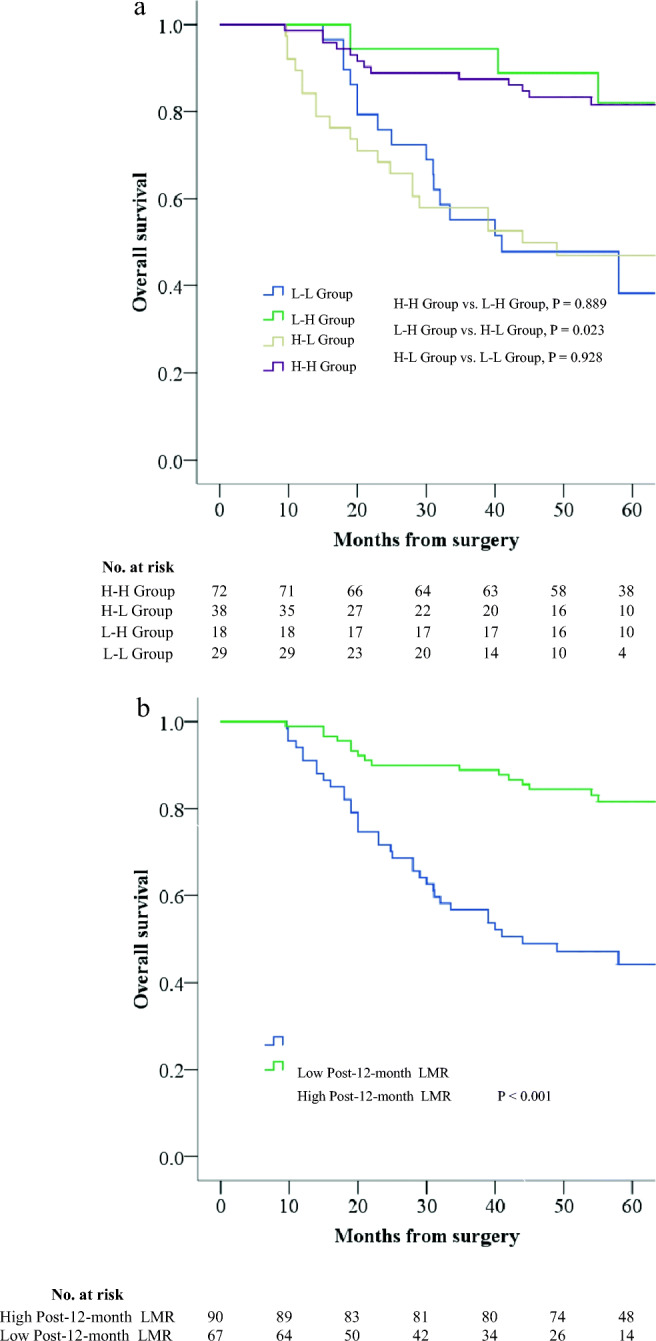
Kaplan-Meier survival curves demonstrating relationship between OS and LMR. **a** Relationship between preoperative and post-12-month LMR change groups and OS. **b** Relationship between post-12-month LMR and OS

Because the pre-LMR is the only CBC-based inflammatory marker that affected OS in the multivariate analysis and post-12-month LMR predicted OS with the highest accuracy (Supplementary Table 2), we further explored the effect of changes between the preoperative and post-12-month LMR on OS. Based on the best cutoff values for pre-LMR and post-12-month LMR, we divided patients into the following four groups: high pre-LMR to high post-LMR (H-H) group, high pre-LMR to low post-LMR (H-L) group, low pre-LMR to high post-LMR (L-H) group, and low pre-LMR to low post-LMR (L-L) group. The median follow-up time of this subgroup was 63 months (range from 10 to 79 months), with a 5-year OS rate of 66.9%. In the univariate analysis, age (*P* = 0.013), tumor location (*P* = 0.037), tumor size (*P* = 0.003), vascular invasion (*P* = 0.003), pTNM stage (*P* < 0.001), adjuvant chemotherapy (*P* = 0.004), and LMR change group (*P* < 0.001) were significant. In the multivariate analysis, tumor location (*P* = 0.009), pTNM stage (*P* < 0.001), and LMR change group (*P* = 0.008) remained significant. Examining the changes in the LMR, patients in H-L group had a HR of 2.945 (95% CI 1.308–6.646) compared to the referent H-H group. The 5-year OS rate for the former group was 43.2% compared with 81.6% for the latter group. Patients in L-H group had an HR of 1.239 (95% CI 0.378–4.056), with a 5-year OS rate of 81.8%. Patients in L-L group had an HR of 3.682 (95% CI 1.607–8.436), with a 5-year OS rate of 45.8% (Table 2; Fig. 2a). Regardless of pre-LMR levels, 5-year OS was significantly higher in patients with high post-12-month LMR than in patients with low post-12-month LMR (81.6% vs. 44.2%, *P* < 0.001) (Fig. 2b).

**Table 2:** Univariate and multivariate analyses of clinicopathological variables in relation to overall survival in the subcohort of patients with bloods available preoperatively and also at post-12-month

Clinicopathological features	Univariate analysis	*P*	Multivariate analysis	*P*
	HR (95% CI)		HR (95% CI)	
Age				
< 60	Reference		Reference	
≥ 60	2.031 (1.161–3.556)	0.013	1.537 (0.824–2.864)	0.176
Sex				
Male	Reference			
Female	0.586 (0.277–1.243)	0.164		
BMI		0.851		
BMI < 18.5	Reference			
18.5 ≤ BMI < 25	1.180 (0.365–3.818)	0.782		
BMI ≥ 25	1.379 (0.384–4.951)	0.622		
ASA score		0.340		
1	Reference			
2	0.868 (0.492–1.531)	0.624		
3	2.168 (0.661–7.117)	0.202		
Tumor location		0.037		0.009
Upper	Reference		Reference	
Middle	0.437 (0.198–0.964)	0.040	0.253 (0.104–0.615)	0.002
Lower	0.417 (0.207–0.840)	0.014	0.307 (0.138–0.680)	0.004
Mixed	0.857 (0.389–1.888)	0.701	0.429 (0.164–1.121)	0.084
Tumor size (mm)				
< 50	Reference		Reference	
≥ 50	2.365 (1.342–4.168)	0.003	0.945 (0.487–1.833)	0.867
Histologic type				
Differentiated	Reference			
Undifferentiated	0.996 (0.523–1.897)	0.989		
Vascular invasion				
Negative	Reference		Reference	
Positive	2.286 (1.336–3.910)	0.003	1.419 (0.732–2.751)	0.300
Perineural invasion				
Negative	Reference			
Positive	1.738 (0.956–3.158)	0.070		
pTNM stage		< 0.001		< 0.001
I	Reference		Reference	
II	5.866 (0.734–46.911)	0.095	6.164 (0.659–57.680)	0.111
III	26.710 (3.678–193.982)	0.001	26.031 (2.850–237.739)	0.004
Adjuvant chemotherapy*				
No	Reference		Reference	
Yes	3.464 (1.474–8.143)	0.004	0.554 (0.198–1.551)	0.261
LMR change group		< 0.001		0.008
High LMR to high LMR	Reference		Reference	
High LMR to low LMR	3.753 (1.892–7.444)	< 0.001	2.948 (1.308–6.646)	0.009
Low LMR to high LMR	1.080 (0.355–3.281)	0.892	1.239 (0.378–4.056)	0.723
Low LMR to low LMR	3.669 (1.782–7.553)	< 0.001	3.682 (1.607–8.436)	0.002

*CI* confidence interval, *HR* hazard ratio, *BMI* body mass index, *ASA* American Society of Anesthesiologists, *TNM* tumor-node-metastasis, *LMR* lymphocyte-monocyte ratio, *NLR* neutrophil-lymphocyte ratio, *PLR* platelet-lymphocyte ratio, *SII* systemic immune-inflammation index

*A total of 12 patients missing adjuvant chemotherapy

We also performed a correlation analysis of post-12-month LMR and recurrence, and the results showed that the low post-12-month LMR group was significantly higher than the high post-12-month LMR group in overall peritoneal and distant recurrence (all *P* < 0.05). But there was no statistical difference in locoregional recurrence between the two groups of patients (Table 3).

**Table 3:** Correlation between post-12-month LMR and recurrence

Site of recurrence	Low LMR (%) *N* = 61*	High LMR (%) *N* = 84*	*P*
Overall	39 (63.9)	24 (28.6)	< 0.001
Locoregional	9 (14.8)	6 (7.1)	0.137
Anastomosis	3 (4.9)	3 (3.6)	1.000
Remnant stomach	2 (3.3)	2 (2.4)	1.000
Regional lymph node	4 (6.6)	1 (1.2)	0.198
Peritoneal	13 (21.3)	4 (4.8)	0.002
Distant	22 (36.1)	15 (17.9)	0.013
Liver	9 (14.8)	7 (8.3)	0.223
Pancreas	0 (0)	1 (1.2)	1.000
Spleen	1 (1.6)	1 (1.2)	1.000
Lung	7 (11.5)	1 (1.2)	0.021
Bone	3 (4.9)	0 (0)	0.143
Distant lymph node	10 (16.4)	10 (11.9)	0.439
Others	4 (6.6)	0 (0)	0.062

*LMR* lymphocyte-monocyte ratio

*A total of 12 patients missing recurrence data

### Clinicopathological Characteristics of Patients with Bloods Available at Post-12-Month by LMR

There were no significant differences in age, sex, BMI, ASA score, tumor location, type of gastrectomy, histologic type, and perineural invasion between high post-12-month LMR and low post-12-month LMR groups. Compared with patients with high post-12-month LMR, patients with low post-12-month LMR had larger tumor size, higher rate of vascular invasion, more advanced pTNM stage, higher rate of adjuvant chemotherapy, higher post-12-month carcinoembryonic antigen (CEA), and post-12-month carbohydrate antigen 19-9 (CA-199) levels (all *P* < 0.05) (Supplementary Table 3).

We also compared the post-12-month LMR levels between patients who received postoperative adjuvant chemotherapy and those who did not. The results showed that post-12-month LMR was significantly lower in patients who received postoperative adjuvant chemotherapy than those who did not (*P* < 0.001). Further stratified analysis based on pTNM stage showed that there was no statistical difference in post-12-month LMR between those who received adjuvant chemotherapy and those who did not in patients with the same stage (Supplementary Table 4).

### Incorporating the Post-12-Month LMR into the TNM Staging System

**Fig. 3:**
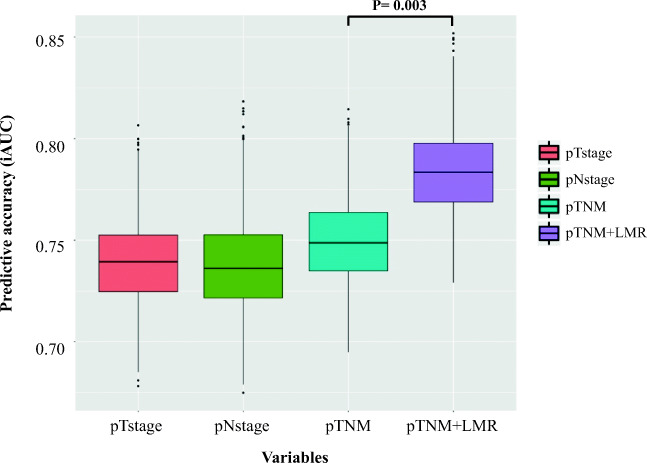
Prognostic performance of post-12-month LMR compared to pathological variables. (The predictive accuracy for 5-year OS based on the iAUC with 1000× bootstrap resampling for each parameter is shown in a box plot)

We utilized the iAUC box plot to represent the AUC with CIs, including pT stage, pN stage, pTNM stage, and post-12-month LMR. As shown in Fig. 3, the pTNM stage was superior to both the pT stage and pN stage for determining the prognosis of patients. However, the combination of the post-12-month LMR and pTNM stage further improved the accuracy of the predicted prognosis determined for these patients, which was better than the TNM stage alone (*P* = 0.003).

## Discussion

This study is the first to show the dynamic changes in the preoperative and post-SIR in patients with GC by performing a retrospective analysis of a large population. The NLR, PLR, and SII in GC returned to preoperative levels at post-12-month, while post-12-month LMR was still significantly higher than preoperative (*P* = 0.007). A pre-LMR ≥ 3.42 was an independent protective factor for the prognosis in patients with GC (HR = 0.810, 95% CI 0.674–0.973, *P* = 0.024). The post-12-month LMR displayed the greatest accuracy in predicting the 5-year OS rate (AUC 0.717, 95% CI 0.631–0.804). A survival analysis combing preoperative and post-12-month LMRs showed that the 5-year OS rate of patients with an LMR ≥ 4.00 was higher than patients with an LMR < 4.00 regardless of pre-LMR level. The incorporation of the post-12-month LMR into the TNM staging system significantly improved the accuracy of the prognosis (*P* = 0.003).

Inflammation is one of the seven characteristics of cancer, causing approximately 25% of new cancer cases worldwide.^19, 20^ Based on accumulating evidence, the inflammatory response can promote tumor development, including the initial stage of tumorigenesis, proliferation, angiogenesis, metastasis, and other processes.^21–23^ LMR, NLR, PLR, and SII are conveniently accessible in the clinic, display high reproducibility, and their dynamic changes are easy to observe. A series of studies have confirmed that pre-LMR, NLR, PLR, and SII are markers of SIR and are closely related to the survival of patients with GC.^24–27^ However, researchers have not studied the changes in SIR levels in patients with GC before and after surgery and their effects on the prognosis. Previous studies have confirmed that the resection margin (R-status) is an independent predictor of OS in patients with GC. Badgwell et al. found that the risk of death with R1 resection was 2.29 comparing to R0 resection (HR 2.29; 95% CI 1.13 to 2.74).^28^ Because the prognosis of patients with R1 and R0 resection is very different, we only included patients with R0 resection in order to investigate the changes of pre- and postoperative SIR and their prognosis values for GC patients received radical gastrectomy.

This study reported the changes in perioperative SIR levels in patients with GC by describing the dynamic changes in LMR, NLR, PLR, and SII recorded at preoperative and postoperative time points. The LMR and NLR were higher at each time point after surgery than at the preoperative assessment, while PLR and SII were lower than the preoperative values. A potential explanation for these findings is that radical surgery eliminates most of the tumor burden along with the hematological effects of chemotherapy. At 12 months after surgery, the pre- and postoperative differences in NLR, PLR, and SII were no longer statistically significant with the end of adjuvant chemotherapy, and the LMR was still significantly higher than the preoperative value. In a previous study, mononuclear cells were described as the key to maintaining chronic inflammatory processes^29^; therefore, the consistently higher post-LMR than the preoperative value may reflect long-term chronic inflammatory processes that do not easily change with the termination of cancer treatment. As shown in our study, neutrophils and platelets appear to be more sensitive to chemotherapy, and these two cell types are often used as indicators to assess the patient’s tolerance to chemotherapy, which is also confirmed by our results. However, a clear understanding of the mechanism underlying these differences was beyond the scope of the current study.

The second part of the study explored the effects of changes in preoperative and post-SIR levels on long-term survival. Because the LMR was the only CBC-based inflammatory marker that affected OS in the multivariate analysis and LMR predicted long-term survival with the highest accuracy at 12 months postoperatively, we combined the pre-LMR and post-12-month LMR to explore the prognostic value of changes in SIR levels. According to X-tile software, the optimal cutoff value for the pre-LMR was 3.42, and the optimal cutoff value for the post-12-month LMR was 4.00. Vincent et al. investigated risk factors for predicting the early recurrence of pancreatic ductal adenocarcinoma. The best predictive cutoff value for preoperative CA19-9 was 210 U/ml, and the optimal cutoff value for postoperative CA-199 was 37 U/ml.^30^ The difference in the optimal threshold of hematological parameters before and after surgery may be caused by surgery-related stress, postoperative complications, and the administration of adjuvant chemotherapy. Patients with a low pre-LMR and low post-LMR exhibited a similar lower 5-year OS rate to patients with a higher pre-LMR and low post-LMR in the present study. Patients with a high pre-LMR and high post-LMR had a similar high 5-year OS rate to patients with a low pre-LMR and high post-LMR. The LMR level at 12 months postoperatively determined the long-term survival of patients, but survival was not affected by pre-LMR levels. This did not mean that pre-LMR is not important. The accuracy of the 5-year overall survival predicted by the pre-LMR is only lower than the post-12-month LMR but higher than other time points post-LMR. These results indicate that pre-LMR has predictive value for prognosis in patients with GC for a period of up to 1 year. However, when the follow-up is up to 12th month after surgery, the LMR obtained by retesting of blood routine examination can replace the pre-LMR as an indicator of prognosis. These findings provide additional prognostic information that was not reported in previous studies examining the pre-LMR alone.^13^

The TNM staging system is a standard method for staging GC used by clinicians and medical workers. It is mainly used for an evaluation of the postoperative prognosis and follow-up treatment decision making processes.^17^ Combined with the findings of this study, we obtained a modified predictive model that was established by incorporating the LMR level at 12 months postoperatively into the TNM staging system, which had a higher AUC than the TNM staging system (*P* = 0.003). Thus, the LMR recorded at 12 months after surgery predicts survival independent of the TNM staging system, and its combined application with the TNM staging system improves the prognostic accuracy.

Our study has some limitations. First, this study is a single-center exploratory study that lacks external validation. Second, because not all patients are scheduled for blood tests after surgery, fewer results were available at 12 months postoperatively. Nevertheless, this study is the first to describe the changes in perioperative SIR levels in patients with GC and to determine their prognostic value. These findings may serve as bases for further prospective studies and may ultimately affect the follow-up strategies for GC patients.

## Electronic Supplementary Material

ESM 1 (PDF 85 kb)

ESM 2 (DOCX 17 kb)

ESM 3 (DOCX 18 kb)

ESM 4 (DOCX 21 kb)

ESM 5 (DOCX 15 kb)

## Data Availability

The datasets used and/or analyzed during the current study are available from the corresponding author upon reasonable request.
