# Supplementary figures and images for: Dynamic Changes in Pre- and Postoperative Levels of Inflammatory Markers and Their Effects on the Prognosis of Patients with Gastric Cancer
Source: J Gastrointest Surg. 2020 Feb 3;25(2):387–96. doi: 10.1007/s11605-020-04523-8 (PMC7904717; doi:10.1007/s11605-020-04523-8)

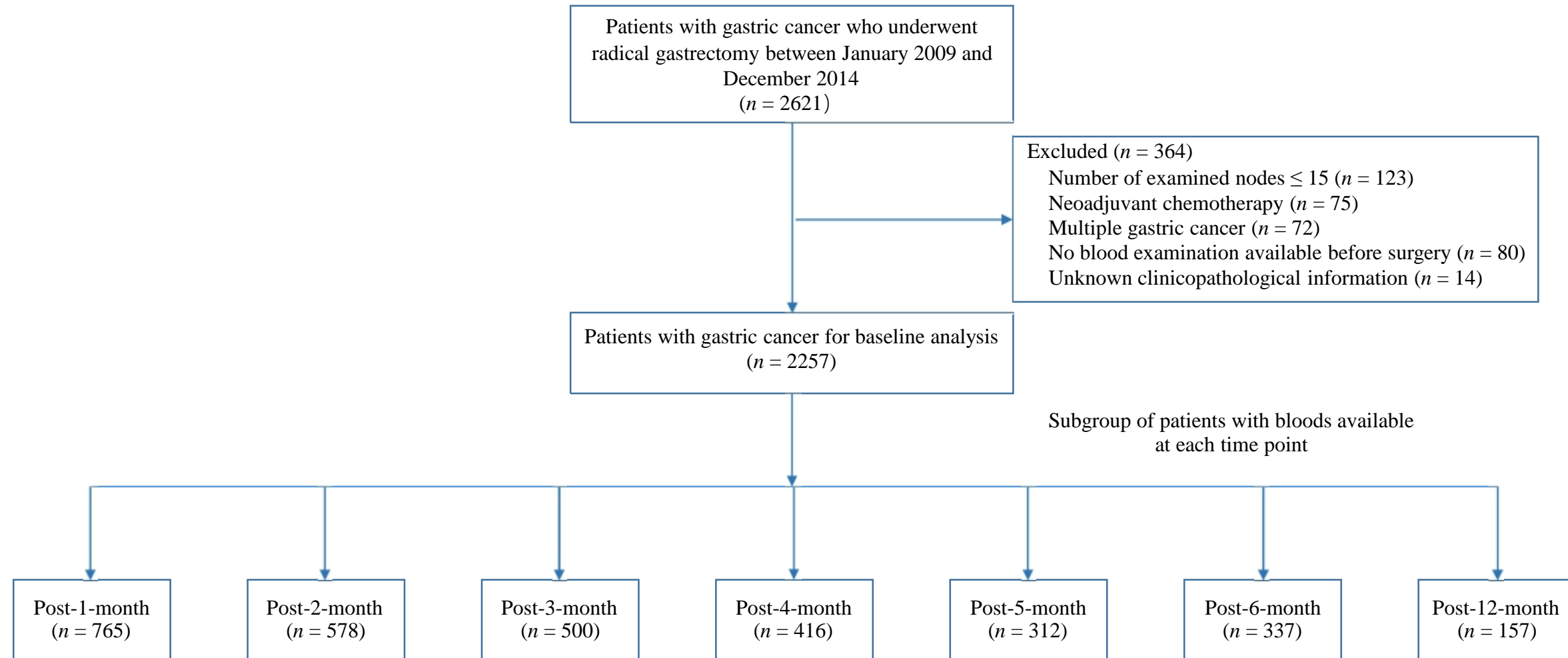

Supplement: Supplementary file 1 — (PDF 85 kb) [file 11605_2020_4523_MOESM1_ESM.pdf]
