# Supplementary material for: Dynamic Changes in Pre- and Postoperative Levels of Inflammatory Markers and Their Effects on the Prognosis of Patients with Gastric Cancer
Source: J Gastrointest Surg. 2020 Feb 3;25(2):387–96. doi: 10.1007/s11605-020-04523-8 (PMC7904717; doi:10.1007/s11605-020-04523-8)
Supplement: Supplementary file 2 — (DOCX 17 kb) [file 11605_2020_4523_MOESM2_ESM.docx]

**Supplementary Table 1.** Baseline Clinicopathological Characteristics of Patients

| Clinicopathological features | All cases = 2257 |
| --- | --- |
| Patient characteristics |  |
| Age, mean ± SD | 60.9 ± 11.2 |
| Sex |  |
| Male | 1698 (75.2) |
| Female | 559 (24.8) |
| BMI, mean ± SD | 22.5 ± 8.8 |
| ASA score |  |
| 1 | 1425 (63.1) |
| 2 | 761 (33.7) |
| 3 | 71 (3.1) |
| Disease characteristics |  |
| Tumor location |  |
| Upper | 540 (23.9) |
| Middle | 490 (21.7) |
| Lower | 950 (42.1) |
| Mixed | 277 (12.3) |
| Type of gastrectomy |  |
| Total | 1180 (52.3) |
| Distal | 1027 (45.5) |
| Proximal | 49 (2.2) |
| Tumor size (mm), mean ± SD | 45.2 ± 25.1 |
| Histologic type |  |
| Differentiated | 517 (22.9) |
| Undifferentiated | 1740 (77.1) |
| Vascular invasion |  |
| Negative | 1741 (77.1) |
| Positive | 516 (22.9) |
| Perineural invasion |  |
| Negative | 1887 (83.6) |
| Positive | 370 (16.4) |
| pTNM stage |  |
| I | 660 (29.2) |
| II | 553 (24.5) |
| III | 1044 (46.3) |
| Adjuvant chemotherapy* |  |
| Yes | 934 (52.7) |
| No | 838 (47.3) |
| CBC-based inflammatory markers |  |
| LMR, mean ± SD | 4.5 ± 2.1 |
| NLR, mean ± SD | 2.6 ± 2.2 |
| PLR, mean ± SD | 154.2 ± 79.1 |
| SII, mean ± SD | 636.6 ± 593.5 |

**Abbreviations:** SD, standard deviation; BMI, body mass index; ASA, American Society of Anesthesiologists; TNM, tumor-node-metastasis; CBC, complete blood count; LMR, lymphocyte-monocyte ratio; NLR, neutrophil-lymphocyte ratio; PLR, platelet-lymphocyte ratio; SII, systemic immune-inflammation index

*485 patients missing Adjuvant chemotherapy
