# Supplementary material for: Dynamic Changes in Pre- and Postoperative Levels of Inflammatory Markers and Their Effects on the Prognosis of Patients with Gastric Cancer
Source: J Gastrointest Surg. 2020 Feb 3;25(2):387–96. doi: 10.1007/s11605-020-04523-8 (PMC7904717; doi:10.1007/s11605-020-04523-8)
Supplement: Supplementary file 3 — (DOCX 18 kb) [file 11605_2020_4523_MOESM3_ESM.docx]

**Supplementary Table 2.** Comparison of the AUCs between CBC-based inflammatory markers at each time period

| Time period | AUC for 5-year OS | | | 95% CI | Time period | | AUC for 5-year OS | | | 95% CI |  |
| --- | --- | --- | --- | --- | --- | --- | --- | --- | --- | --- | --- |
| LMR | |  |  | | | PLR | |  |  | | |
| Preoperative | | 0.572 | 0.574 - 0.598 | | | Preoperative | | 0.579 | 0.553 - 0.604 | | |
| Postoperative | |  |  | | | Postoperative | |  |  | | |
| 1 month | | 0.526 | 0.482 - 0.571 | | | 1 month | | 0.508 | 0.463 - 0.553 | | |
| 2 month | | 0.535 | 0.483 - 0.587 | | | 2 month | | 0.508 | 0.456 - 0.561 | | |
| 3 month | | 0.551 | 0.496 - 0.605 | | | 3 month | | 0.514 | 0.456 - 0.572 | | |
| 4 month | | 0.539 | 0.476 - 0.601 | | | 4 month | | 0.582 | 0.521 - 0.643 | | |
| 5 month | | 0.563 | 0.490 - 0.637 | | | 5 month | | 0.561 | 0.501 - 0.641 | | |
| 6 month | | 0.568 | 0.493 - 0.641 | | | 6 month | | 0.508 | 0.439 - 0.577 | | |
| 12 month | | **0.717** | **0.631 - 0.804** | | | 12 month | | 0.553 | 0.451 - 0.654 | | |
| NLR | |  |  | | | SII | |  |  | | |
| Preoperative | | 0.558 | 0.574 - 0.598 | | | Preoperative | | 0.558 | 0.574 - 0.598 | | |
| Postoperative | |  |  | | | Postoperative | |  |  | | |
| 1 month | | 0.504 | 0.460 - 0.549 | | | 1 month | | 0.504 | 0.459 - 0.549 | | |
| 2 month | | 0.505 | 0.454 - 0.556 | | | 2 month | | 0.503 | 0.450 - 0.555 | | |
| 3 month | | 0.511 | 0.454 - 0.568 | | | 3 month | | 0.515 | 0.457 - 0.572 | | |
| 4 month | | 0.539 | 0.478 - 0.600 | | | 4 month | | 0.545 | 0.485 - 0.606 | | |
| 5 month | | 0.509 | 0.435 - 0.583 | | | 5 month | | 0.529 | 0.454 - 0.603 | | |
| 6 month | | 0.525 | 0.457 - 0.593 | | | 6 month | | 0.527 | 0.458 - 0.595 | | |
| 12 month | | 0.600 | 0.501 - 0.699 | | | 12 month | | 0.567 | 0.467 - 0.668 | | |

**Abbreviations:** AUC, the areas under the curve; CBC, complete blood count; OS, overall survival; LMR, lymphocyte-monocyte ratio; NLR, neutrophil-lymphocyte ratio; PLR, platelet-lymphocyte ratio; SII, systemic immune-inflammation index
