# Supplementary material for: Dynamic Changes in Pre- and Postoperative Levels of Inflammatory Markers and Their Effects on the Prognosis of Patients with Gastric Cancer
Source: J Gastrointest Surg. 2020 Feb 3;25(2):387–96. doi: 10.1007/s11605-020-04523-8 (PMC7904717; doi:10.1007/s11605-020-04523-8)
Supplement: Supplementary file 4 — (DOCX 21 kb) [file 11605_2020_4523_MOESM4_ESM.docx]

**Supplementary Table 3.** Clinicopathological characteristics of patients with bloods available at post-12- month by LMR

| Clinicopathological features | Post-12-month LMR < 4 | Post-12-month LMR ≥ 4 | P |
| --- | --- | --- | --- |
| Age, mean ± SD | 60.2 ± 8.9 | 58.3 ± 10.0 | 0.224 |
| Sex |  |  | 0.127 |
| Male | 56 (83.6) | 66 (73.3) |  |
| Female | 11 (16.4) | 24 (26.7) |  |
| BMI, mean ± SD | 22.5±2.5 | 22.5 ± 3.1 | 0.962 |
| ASA score |  |  | 0.095 |
| 1 | 36 (53.7) | 55 (61.1) |  |
| 2 | 31 (46.3) | 31 (34.4) |  |
| 3 | 0 (0) | 4 (4.4) |  |
| Tumor location |  |  | 0.197 |
| Upper | 9 (13.4) | 18 (20.0) |  |
| Middle | 19 (28.4) | 20 (22.2) |  |
| Lower | 26 (38.8) | 43 (47.8) |  |
| Mixed | 13 (19.4) | 9 (10.0) |  |
| Type of gastrectomy |  |  | 0.560 |
| Total | 37 (55.2) | 42 (46.7) |  |
| Distal | 29 (43.3) | 46 (51.1) |  |
| Proximal | 1 (1.5) | 2 (2.2) |  |
| Tumor size (mm), mean ± SD | 54.2 ± 25.0 | 44.8 ± 27.9 | 0.030 |
| Histologic type |  |  | 0.409 |
| Differentiated | 12 (17.9) | 21 (23.3) |  |
| Undifferentiated | 55 (82.1) | 69 (76.7) |  |
| Vascular invasion |  |  | 0.036 |
| Negative | 41 (61.2) | 69 (76.7) |  |
| Positive | 26 (38.8) | 21 (23.3) |  |
| Perineural invasion |  |  | 0.189 |
| Negative | 51 (76.1) | 76 (84.4) |  |
| Positive | 16 (23.9) | 14 (15.6) |  |
| pTNM stage |  |  | < 0.001 |
| I | 3 (4.5) | 29 (32.2) |  |
| II | 18 (26.9) | 27 (30.0) |  |
| III | 46 (68.7) | 34 (37.8) |  |
| Adjuvant chemotherapy* |  |  | < 0.001 |
| Yes | 53 (93.0) | 38 (43.2) |  |
| No | 4 (7.0) | 50 (56.8) |  |
| CEA (ng/mL), mean ± SD** | 10.2 ± 21.3 | 3.5 ± 4.0 | 0.029 |
| CA19-9 (U/mL), mean ± SD*** | 98.1 ± 242.0 | 12.9 ± 20.3 | 0.014 |

**Abbreviations:** SD, standard deviation; BMI, body mass index; ASA, American Society of Anesthesiologists; TNM, tumor-node-metastasis; LMR, lymphocyte-monocyte ratio; CEA, carcinoembryonic antigen; CA19-9, carbohydrate antigen 19-9

*12 patients missing Adjuvant chemotherapy

**23 patients missing CEA

***21 patients missing CA19-9
