# Supplementary material for: Dynamic Changes in Pre- and Postoperative Levels of Inflammatory Markers and Their Effects on the Prognosis of Patients with Gastric Cancer
Source: J Gastrointest Surg. 2020 Feb 3;25(2):387–96. doi: 10.1007/s11605-020-04523-8 (PMC7904717; doi:10.1007/s11605-020-04523-8)
Supplement: Supplementary file 5 — (DOCX 15 kb) [file 11605_2020_4523_MOESM5_ESM.docx]

**Supplementary Table 4.** Post-12- month LMR levels by chemotherapy and TNM stage

| Post-12- month LMR levels | Chemotherapy | No Chemotherapy | P |
| --- | --- | --- | --- |
| All 157 patients | 6.3 ± 2.1 | 4.9 ± 2.9 | 0.003 |
| Stage I | 6.6 ± 2.2 | 8.8 ± 4.6 | 0.221 |
| Stage II | 5.9 ± 0.8 | 5.4 ± 3.4 | 0.721 |
| Stage III | 5.6 ± 2.0 | 4.4 ± 2.3 | 0.205 |

**Abbreviations:** LMR, lymphocyte-monocyte ratio; TNM, tumor-node-metastasis
